# Clinical Implications of Ivabradine in the Contemporary Era

**DOI:** 10.3390/medicina60020303

**Published:** 2024-02-10

**Authors:** Teruhiko Imamura

**Affiliations:** The Second Department of Internal Medicine, University of Toyama, Toyama 930-8555, Japan; teimamu@med.u-toyama.ac.jp; Tel.: +81-76-434-2246; Fax: +81-76-434-5026

**Keywords:** heart failure, hemodynamics, arrhythmia, beta-blocker

## Abstract

Ivabradine is a recently introduced inhibitor of the I_f_ ion channel, which exhibits the capacity to reduce heart rate while preserving hemodynamic stability. At present, ivabradine finds its clinical indication in patients suffering from heart failure with reduced ejection fraction and maintaining a relative sinus rhythm refractory to beta-blockers. To optimize heart rate control, it is recommended to pursue an aggressive up-titration of ivabradine. This approach may ameliorate tachycardia-induced hypotension by incrementally enhancing cardiac output and allow further up-titration of agents aimed at ameliorating heart failure, such as beta-blockers. Both the modulation of heart rate itself and the up-titration of agents targeting heart failure lead to cardiac reverse remodeling, consequently culminating in a subsequent reduction in mortality and morbidity. A novel overlap theory that our team proposed recently has emerged in recent times. Under trans-mitral Doppler echocardiography, the E-wave and A-wave closely juxtapose one another without any overlapping at the optimal heart rate. Employing echocardiography-guided ivabradine for heart-rate modulation to minimize the overlap between the E-wave and A-wave appears to confer substantial benefits to patients with heart failure. This approach facilitates superior cardiac reverse remodeling and yields more favorable clinical outcomes when compared to those patients who do not receive echocardiography-guided care. The next pertinent issue revolves around the potential expansion of ivabradine’s clinical indications to encompass a broader spectrum of diseases. It is imperative to acknowledge that ivabradine may not yield clinically significant benefits in patients afflicted by heart failure with preserved ejection fraction, acute heart failure, sepsis, or stable angina. An important fact yet to be explored is the clinical applicability of ivabradine in patients with atrial fibrillation, a concern that beckons future investigation. In this review, the concept of overlap theory it introduced, along with its application to expand the indication of ivabradine and the overlap theory-guided optimal ivabradine therapy.

## 1. Introduction: How Heart Rate Modulation Is Important

In recent years, a new class of neuro-hormonal blockers, encompassing beta-blockers, renin-angiotensin-aldosterone system inhibitors, and sodium-glucose co-transporter 2 inhibitors, has made significant strides in improving the prognosis and quality of life for patients afflicted by chronic heart failure with reduced ejection fraction (HFrEF) [1,2,3]. Despite these advancements, patient outcomes have not yet reached the desired levels due to lingering cardiovascular risks.

One of the unmet therapeutic objectives centers on heart-rate management [4]. A comprehensive analysis of Swedish registry data, featuring patients suffering from HFrEF and maintaining a sinus rhythm (as opposed to atrial fibrillation), has revealed a robust correlation between a lower heart rate and a reduction in all-cause mortality [5]. It has been postulated that a lower heart rate is intricately linked to enhanced cardiac output, heightened success in cardiac reverse remodeling, and superior clinical outcomes [6].

Ivabradine, a recently introduced I_f_ ion channel blocker, has emerged as a promising solution for heart-rate control, all the while preserving hemodynamic stability [7]. In alignment with the aforementioned hypothesis, the SHIFT trial was conducted [8,9]. This clinical investigation showcased that ivabradine, when compared to a placebo, yielded a notable increase in freedom from death or heart-failure recurrence among patients with HFrEF.

However, the optimal target heart rate during ivabradine therapy remains a subject of concern. Subsequent analysis of the SHIFT trial unveiled that an excessively aggressive reduction in heart rate paradoxically elevated the risk of cardiovascular events [10]. In response to this challenge, the author’s team has recently introduced an innovative method to determine the ideal target heart rate for each patient, employing trans-mitral Doppler echocardiography as a reference [11]. This method’s applicability has been explored across a spectrum of clinical scenarios, including heart failure with preserved ejection fraction (HFpEF) [12].

Furthermore, the application of ivabradine therapy has been extended to patients with other medical conditions, such as atrial fibrillation [13]. In this age of medical advancement, clinicians must engage in a comprehensive examination of ivabradine’s position among these treatment modalities, sometimes referred to as the “fantastic four.” This review aims to provide an updated perspective on ivabradine therapy in the contemporary medical landscape.

## 2. Beta-Blocker as a Mainstay for HFrEF and Challenges in Its Up-Titration

The therapeutic landscape for patients with HFrEF has undergone substantial refinement over the years. Contemporary guidelines emphasize the pivotal role of beta-blockers alongside other neuro-hormonal antagonists, such as renin–angiotensin–aldosterone system inhibitors and sodium–glucose co-transporter 2 inhibitors, in the management of HFrEF [1,2,3]. Much like their counterparts, the optimal strategy for beta-blockers necessitates diligent dose escalation to ameliorate heart-failure symptoms, foster cardiac reverse remodeling, and mitigate the risk of sudden cardiac death [14]. Of note, beta-blocker is a major therapeutic option for heart-rate modulation [15].

**Figure 1:**
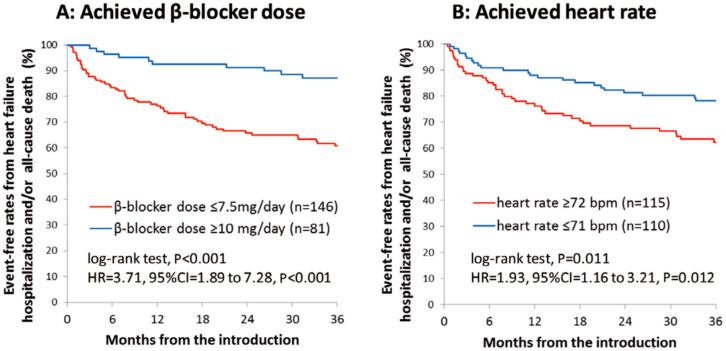
Event-free rates from heart-failure hospitalization or all-cause death stratified by the achievement of beta-blocker doses equal to or above 10 mg/day or not (**A**) and by the achievement of resting heart rate equal to or above 72 beats per minute (**B**) in a large academic center (with permission to reuse from the publisher) [16]. Patients with a lower dose of beta-blockers or those with higher heart rate had lower freedom from the composite endpoints. HR, hazard ratio; CI, confidence interval. Kaplan–Meier curves were compared by log-rank test.

**Figure 2:**
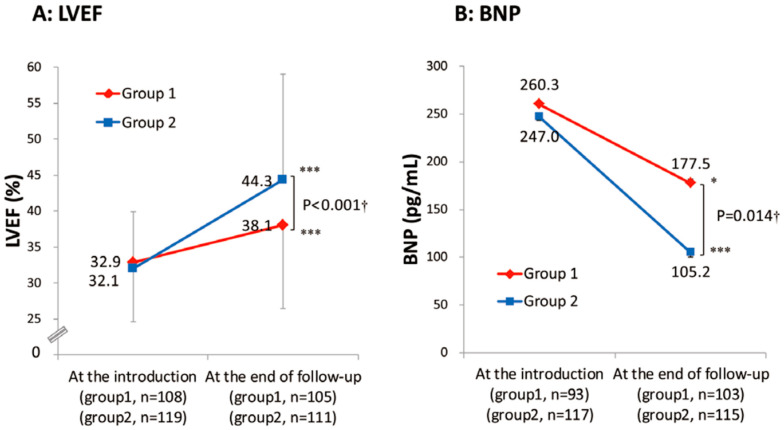
Trends of left-ventricular ejection fraction (**A**) and plasma B-type natriuretic peptide levels (**B**) in the two groups stratified by the study period with different up-titration policies (with permission to reuse from the publisher) [16]. Group 1 was defined as patients who received beta-blockers before December 2005. Group 2 was defined as patients who received beta-blockers after December 2005. Group 1 had insufficient improvement in LVEF and plasma BNP levels during the observation period compared with Group 2. † Unpaired *t*-test. *** *p* <0.001 and * *p* < 0.05 by paired *t*-test.

However, the real-world application of beta-blocker up-titration often presents formidable challenges. Within a prominent Japanese academic medical center, it was observed that nearly two-thirds of HFrEF patients received less than half of the recommended target beta-blocker dose [16]. Notably, this suboptimal dosing regimen was associated with a significantly diminished three-year freedom from cardiac mortality when compared to their adequately treated counterparts (Figure 1A) [16]. Furthermore, individuals characterized by a heart rate equal to or exceeding 72 beats per minute experienced notably lower rates of freedom from cardiac mortality (Figure 1B) [16]. Patients with lower beta-blocker doses experienced an inferior improvement in left-ventricular ejection fraction and less improvement in plasma B-type natriuretic peptide levels compared to their counterparts (Figure 2A,B) [16]. Thus, insufficient up-titration of beta-blockers is one of the unmet needs in contemporary real-world heart-failure management.

In everyday clinical practice, a range of clinical scenarios are linked to challenges in achieving the desired up-titration of beta-blockers. Notably, refractoriness to dose escalation may be associated with unresolved congestion and persistent hypotension [17]. In instances of decompensated congestive heart failure, inappropriately aggressive beta-blocker up-titration can exacerbate pulmonary congestion, thus exacerbating patients’ clinical status. Similarly, the vigorous up-titration of beta-blockers in individuals experiencing hypotension can further compromise their blood pressure, necessitating a careful and individualized approach to therapy.

## 3. Indication for Ivabradine

As elucidated in the introductory section, the management of heart-rate modulation stands as a paramount therapeutic objective for individuals suffering from HFrEF [4]. However, the limitations associated with beta-blockers, as described above, render them occasionally insufficient in effectively addressing tachycardia. In this context, the introduction of ivabradine offers a valuable alternative for heart-rate reduction, one that leaves hemodynamics unaltered. Ivabradine is especially well-suited for patients who prove refractory to further beta-blocker up-titration [18]. It is recommended in cases where beta-blockers cannot be escalated despite the persistence of a relatively elevated heart rate within a sinus rhythm. Importantly, the administration of ivabradine need not wait until the beta-blocker has reached its maximum target dose.

Indications of ivabradine for heart-failure patients are stated in each guideline (Table 1). Within the framework of the Japanese guidelines, ivabradine is indicated for patients maintaining sinus rhythm, with a heart rate equal to or surpassing 75 beats per minute and a left-ventricular ejection fraction equal to or less than 35% [1]. This recommendation holds even when adhering to guideline-directed medical therapy, including maximizing beta-blockers as tolerated, garnering a class IIa recommendation. The guidelines set forth by the European Society of Cardiology designate ivabradine as a class IIa recommendation for individuals with normal sinus rhythm, a left-ventricular ejection fraction equal to or less than 40%, and a heart rate equal to or surpassing 70 beats per minute, rather than 75 beats per minute [2]. This indication aligns closely with the guidelines outlined by the American Heart Association and underscores the consistent recognition of ivabradine’s clinical utility [3].

## 4. The SHIFT Trial: A Cornerstone Trial That Established the Current Indication of Ivabradine

**Figure 3:**
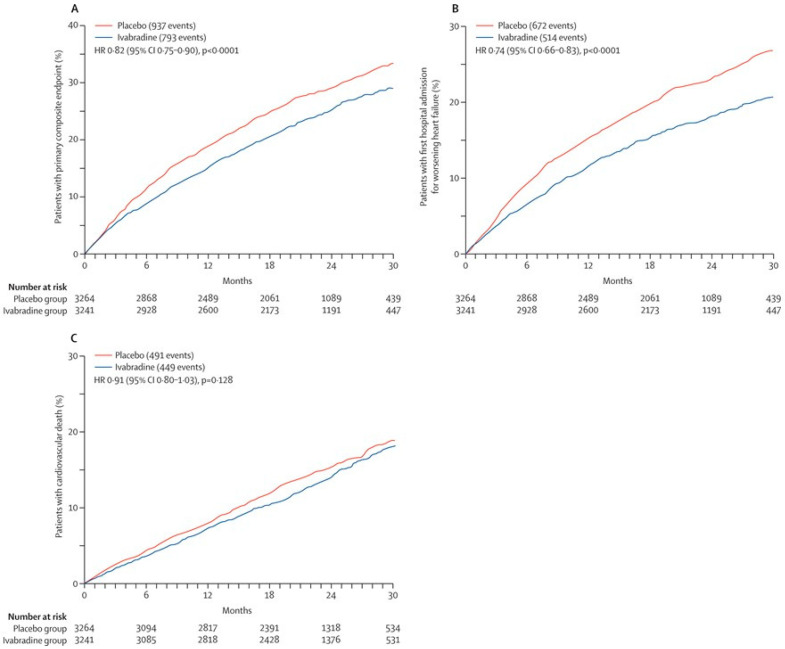
Comparison in the cumulative incidence of the primary composite endpoint (**A**), first hospitalization for worsening heart failure (**B**), and cardiovascular death (**C**) between the ivabradine arm and the placebo arm in the SHIFT trial (with permission to reuse from the publisher) [8]. The ivabradine group had a lower cumulative incidence of each event, whereas the difference in cardiovascular death did not reach statistical significance. HR, hazard ratio. Kaplan–Meier curves were compared by log-rank test.

The foundation of these guidelines is rooted in the seminal SHIFT trial [8,9]. This pivotal clinical investigation encompassed patients characterized by sinus rhythm, a left-ventricular ejection fraction equal to or less than 35%, and a heart rate equal to or exceeding 70 beats per minute [19]. These individuals were randomized into two groups: one receiving ivabradine and the other a placebo. The analysis revealed a substantial reduction in the cumulative incidence of cardiovascular death or heart-failure readmission in the ivabradine group, exhibiting a noteworthy hazard ratio of 0.82 (Figure 3A–C) [8]. The beneficial effects of ivabradine were evident irrespective of the severity of heart-failure symptoms, as defined by the New York Heart Association functional class. Thus, the SHIFT trial is a cornerstone of ivabradine therapy for individuals with higher heart rate, sinus rhythm, and reduced ejection fraction.

In a sub-analysis of the SHIFT trial, ivabradine therapy yielded modest cardiac reverse remodeling [20]. This entailed a reduction of 5.5 mL/m^2^ in left-ventricular end-diastolic volume index and a concurrent increase of 2.7% in left-ventricular ejection fraction. Notably, those patients who achieved substantial reverse remodeling experienced a significantly reduced cumulative incidence of cardiac death or heart-failure readmission.

## 5. J-SHIFT Trial

In Japan, a parallel study akin to the pivotal phase III SHIFT trial was conducted, known as the J-SHIFT trial [21]. The inclusion and exclusion criteria for this study closely mirrored those of the preceding SHIFT trial, with a notable exception: the threshold for baseline heart rate was set at or above 75 beats per minute, as per the insights gleaned from the SHIFT trial. In the SHIFT trial, patients with a baseline heart rate at or above 75 beats per minute derived greater benefits from ivabradine therapy than their counterparts with a heart rate between 70 and 75 beats per minute [10].

**Figure 4:**
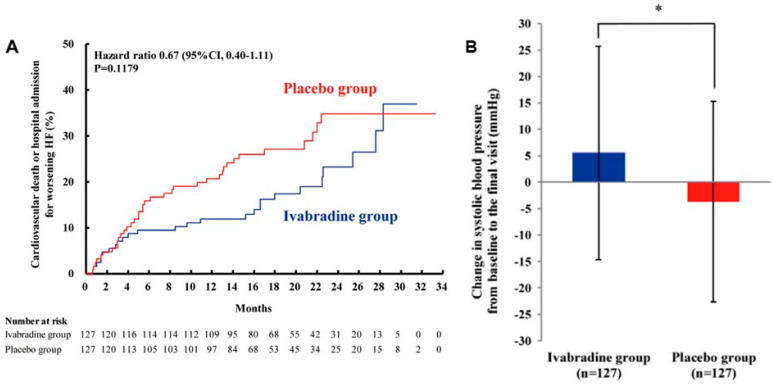
Prospective randomized control trial to compare ivabradine and placebo on the cumulative incidence of cardiovascular death or heart-failure rehospitalization in the J-SHIFT trial (**A**). Change in systolic blood pressure from baseline to the final visit between the ivabradine group vs. the placebo group (**B**) (with permission to reuse from the publisher) [21]. The ivabradine group tended to have lower cumulative incidence of the composite endpoint with preserved systolic blood pressure during the observation period as compared to the placebo group. CI, confidence interval. Log-rank test was used to compare Kaplan–Meier curves. * *p* < 0.05 by unpaired *t*-test.

Throughout the observation period involving ivabradine, a mean heart-rate reduction of 15.2 beats per minute was achieved, as opposed to the 6.1 beats per minute observed in the placebo group. The hazard ratio for ivabradine therapy in comparison to placebo, concerning the cumulative incidence of cardiovascular death or heart-failure readmission, was 0.67, with a 95% confidence interval between 0.40 and 1.11 (Figure 4A) [21]. The indication of ivabradine in Japan has been established according to the findings of this trial.

## 6. Ivabradine and Cardiac Output

The J-SHIFT trial shed light on a noteworthy observation: during ivabradine therapy, a mean increase of 5.5 mmHg in systolic blood pressure was documented, in stark contrast to the −3.7 mmHg decrease observed in the placebo group (Figure 4B) [21]. This phenomenon is likely attributed to the augmentation of cardiac output. This hemodynamic stabilization had a cascading effect, enabling further up-titration of beta-blockers (and other heart-failure medications) [18]. It is well-established that a longer heart period, synonymous with a lower heart rate, is primarily linked to an extension of diastolic time, as opposed to systolic time [22]. Consequently, a reduction in heart rate results in an increased proportion of the cardiac cycle devoted to the diastolic phase. This, in turn, affords heart-failure patients a more substantial diastolic period, which translates to an enhanced cardiac output. Experimental animal studies have also corroborated this phenomenon, demonstrating that ivabradine administration can uphold cardiac output levels despite a reduction in heart rate [23].

## 7. Lower Is Better?

Within the realm of HFrEF patients, it is unequivocally established that sinus tachycardia is intricately linked to heightened mortality and morbidity [24]. Ivabradine offers a tangible clinical advantage by effectively lowering heart rate in this patient cohort. It is imperative to underscore the significance of a judicious approach to ivabradine up-titration, aiming for a substantial reduction in heart rate [25].

The impending question pertains to how far clinicians should lower a patient’s heart rate. It is crucial to recognize that excessively lowering the heart rate may inadvertently diminish cardiac output, characterized by a reduction in the summation of all stroke volumes per minute, potentially culminating in a deteriorated hemodynamic state reminiscent of sick sinus syndrome. In the sub-analysis of the SHIFT trial, it becomes evident that an aggressive reduction in heart rate was warranted to achieve a discernible prognostic benefit among patients entering the trial with a baseline heart rate equal to or exceeding 75 beats per minute [10]. This rationale underscores the importance of advocating for a vigorous up-titration of ivabradine in these cases [25].

Conversely, in patients commencing the trial with a baseline heart rate below 75 beats per minute, a notably aggressive heart-rate reduction paradoxically trended towards an elevated risk of cardiovascular mortality [10]. The data suggest that pushing for extreme heart-rate reduction through ivabradine in such cases may instead exacerbate hemodynamic instability and lead to a deterioration in patient prognosis. How can clinicians estimate the target heart rate in each individual?

## 8. How to Estimate Target Heart Rate

**Figure 5:**
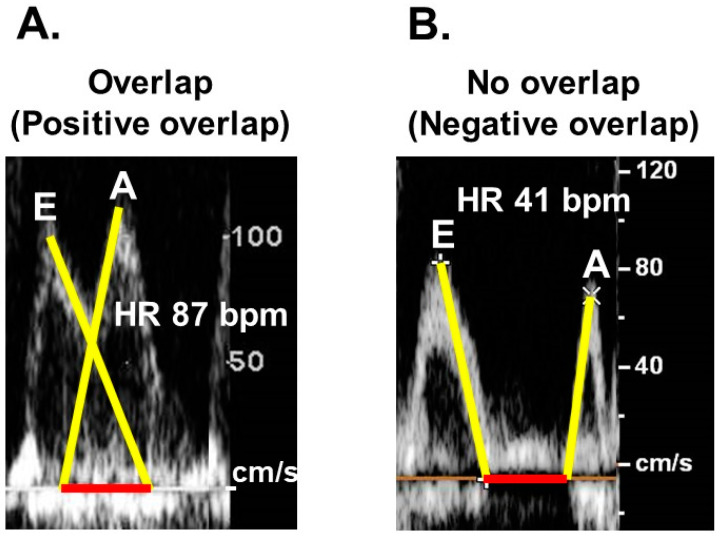
Trans-mitral Doppler echocardiography in patients with an obvious overlap (positive overlap) between the E-wave and A-wave due to a relatively higher heart rate (**A**) and in patients with no overlap (negative overlap) between the two waves due to a relatively lower heart rate (**B**) (with permission to reuse from the publisher) [11]. The obvious wave-overlap indicates insufficient left-ventricular filling time and results in lower cardiac output. The obvious wave-separation indicates sufficient left-ventricular filling time but results also in lower cardiac output due to lower stroke number.

Our research team has put forth a novel approach involving trans-mitral Doppler echocardiography to estimate the optimal target heart rate for each patient (Figure 5A,B) [11]. The trans-mitral flow velocity profile consists of two discernible waves: the E-wave, representing the period of rapid ventricular filling, and the A-wave, denoting the atrial contraction phase [26]. Our hypothesis posits that the presence of overlap between these two waves, a phenomenon commonly observed in patients with sinus tachycardia, signifies an inadequate diastolic filling time, ultimately resulting in diminished cardiac output (Figure 5A) [11]. Conversely, in patients with an excessively low heart rate, the E-wave and A-wave remain distinctly separated, permitting an extended filling duration (Figure 5B) [11]. While this may lead to increased stroke volume, the net effect on cardiac output, which is a summation of all stroke volumes per minute, could be a reduction.

**Figure 6:**
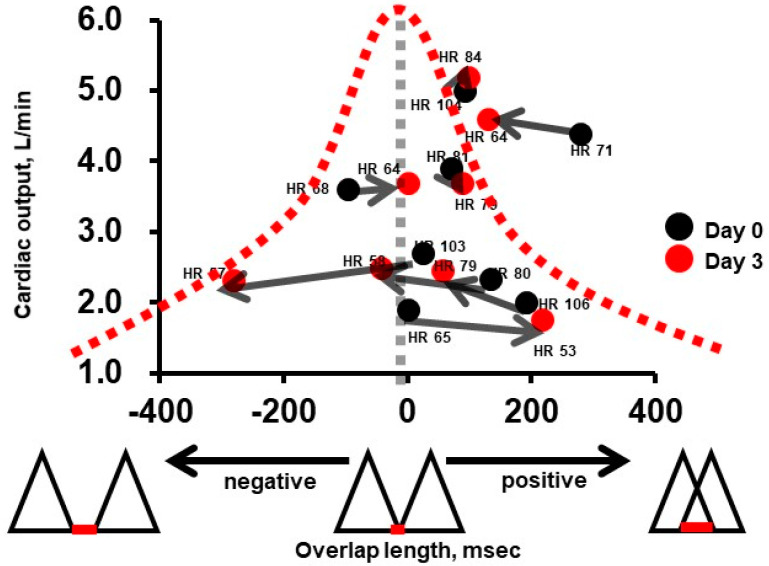
Association between the overlap length and cardiac output in patients receiving 3-day ivabradine therapy (reused as an open license) [27]. Black dots represent day 0 (just before ivabradine therapy) and red dots represent day 3 (3 days after the initiation of ivabradine therapy). Some patients experienced increases in their cardiac output during ivabradine therapy when their overlap length reached zero. Other patients experienced decreases in their cardiac output during ivabradine therapy when their overlap length went negative due to an excessive decline in their heart rate.

Our hypothesis further suggests that the optimal heart rate should correspond with an absence of overlap between the E-wave and A-wave, indicative of an ideal filling duration. This configuration should maximize cardiac output. Our hypothesis was validated through both single and multiple case analyses (Figure 6) [27]. In practice, cardiac output increased when the overlap between the E-wave and A-wave was minimized during ivabradine therapy. In contrast, the separation of these waves during ivabradine therapy (by extreme reduction in heart rate) resulted in a reduction in cardiac output.

Recognizing the potential practical challenges in routinely conducting Doppler echocardiography repeatedly, the author’s team has devised a formula to estimate the ideal heart rate by utilizing the deceleration time [11]:

(ideal heart rate [bpm]) = 93 − 0.13 × (deceleration time [msec])

This formula was generated through a linear regression that integrated heart rate and deceleration time, based on measurements obtained from 368 patients with HFrEF. At the ideal heart rate, the overlap length should ideally reach zero. Consequently, the formula presented above is applicable. For example, when the deceleration time measures 250 milliseconds, this formula calculates the ideal heart rate for the patient as 60 bpm. Interestingly, the ideal heart rate may be unique in each individual.

## 9. Generalization of the Formula to Diverse Clinical Scenarios with HFrEF

The concept of an ideal heart rate, optimized to achieve maximal cardiac output, may hold broader implications beyond HFrEF patients. The next imperative pertains to the prognostic implications of this ideal heart rate. In a prior study involving HFrEF patients, those who maintained heart rates within ±10 beats per minute of their calculated ideal heart rate experienced a significant enhancement in left-ventricular ejection fraction [28]. In stark contrast, individuals with heart rates either lower or higher than the optimal range failed to manifest substantial cardiac reverse remodeling. This observation corroborates our hypothesis, indicating that both excessively high and excessively low heart rates may impede the progress of cardiac reverse remodeling.

This concept extends to other clinical scenarios. For instance, patients grappling with low-flow low-gradient aortic stenosis often contend with persistent systolic heart failure even following trans-catheter aortic valve replacement. In most cases, the left-ventricular ejection fraction tends to improve post-procedure, primarily due to the considerable reduction in afterload occasioned by the relief of aortic valve stenosis. Notably, those individuals with optimal heart rates, defined as falling within 10 beats per minute of the calculated ideal heart rate at the time of their initial discharge following the valve replacement, experienced a more substantial improvement in left-ventricular ejection fraction (i.e., cardiac reverse remodeling) when compared to those with suboptimal heart rates [29]. Thus, the concept of overlap theory seems to be applicable to individuals with low-flow low-gradient aortic stenosis receiving a trans-catheter aortic valve replacement.

In the context of durable left-ventricular assist devices such as the HeartMate 3 [30], which serve as a robust mechanical circulatory support for patients with stage D heart failure, recent studies have shed light on the prospect of cardiac reverse remodeling through mechanical unloading [31]. This transformation in cardiac function is closely associated with several factors, including the reopening of the aortic valve, prevention of aortic insufficiency, mitigation of secondary pulmonary hypertension, and the amelioration of right-ventricular failure [32]. Consequently, aggressive up-titration of neuro-hormonal blockers is a common strategy during mechanical circulatory support [33]. In addition to these interventions, the maintenance of an optimal heart rate at the time of initial discharge following device implantation may be linked to a reduced cumulative incidence of death or heart-failure recurrence, underscoring the possibility of broad applicability of the concept beyond HFrEF patients receiving medical therapy alone [34]. Thus, the concept of overlap theory seems to be applicable also in patients receiving durable left-ventricular assist devices.

## 10. Optimal Heart Rate in HFpEF

The question of establishing the optimal heart rate for patients with HFpEF necessitates distinct considerations. In theory, extending the filling period may not necessarily translate to augmented stroke volume in individuals with diastolic dysfunction [35]. Instead, these patients may benefit from maintaining a relatively higher heart rate to enhance their cardiac output.

**Figure 7:**
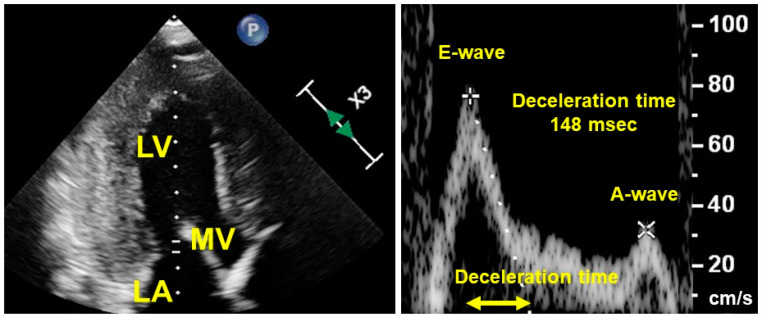
A representation of trans-mitral Doppler echocardiography, presenting a restrictive pattern consisting of a high and narrow E-wave and a small A-wave. LV, left ventricle; MV, mitral valve; LA, left atrium. The patient had heart failure with preserved ejection fraction due to cardiac amyloidosis. The deceleration time of this patient was short. A relatively higher heart rate may be required for both waves to stand adjacent and maximize cardiac output.

Within the context of trans-mitral Doppler echocardiography, patients with diastolic dysfunction often present with a constrictive pattern characterized by a tall and narrow E-wave in conjunction with a diminutive A-wave (see Figure 7 as an example). As per the overlap theory, maintaining a relatively higher heart rate becomes crucial to prevent the E-wave and A-wave from excessively separating [12].

A notable study in the past applied the overlap theory to patients with constrictive pericarditis, a common etiology of diastolic dysfunction [36]. Notably, the median heart rate for individuals in the optimal heart rate group stood at 68 beats per minute, which is relatively higher than the target heart rate for the individuals with HFrEF (typically between 50 and 60 beats per minute). It was observed that patients with heart rates exceeding the optimal range had a higher rate of heart-failure recurrence. Intriguingly, those with lower heart rates (with an actual mean heart rate of 62 bpm) also displayed an elevated incidence of heart-failure recurrence.

Cardiac amyloidosis, another significant contributor to HFpEF, echoes a similar pattern [37]. Per the overlap theory, the calculated ideal heart rate for this patient population demonstrated a median value of 69 beats per minute, once again surpassing the typical values observed in HFrEF cases. Consistent with the findings in constrictive pericarditis, not only higher heart rates but also lower heart rates were associated with a greater incidence of adverse events within this cohort. These observations underscore the nuanced considerations surrounding heart-rate management in patients with HFpEF.

## 11. Echocardiography-Guided Heart-Rate Modulation

The practical application of the overlap theory in guiding heart-rate modulation therapy using ivabradine, regarding trans-mitral Doppler echocardiography, was explored in a recent retrospective analysis conducted by our research team [38]. This analysis aimed to compare the extent of cardiac reverse remodeling between two groups: one in which heart-rate modulation using ivabradine was guided by echocardiography (the echo-guided group) and another in which heart-rate modulation incorporated ivabradine without echocardiographic guidance (the non-echo-guided group), focusing on the HFrEF patient cohort.

**Figure 8:**
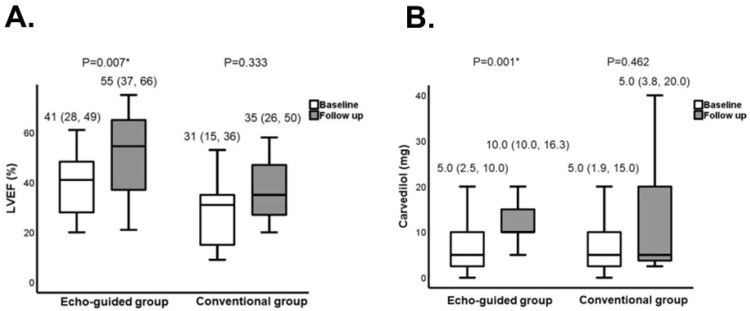
Comparison in clinical parameters between the echo-guided group and conventional group. LVEF (**A**) and the daily dose of carvedilol (**B**) were compared (reused with a permission from the publisher) [38]. Both groups received ivabradine. In an echo-guided group, the dose of ivabradine was adjusted to achieve the optimal heart rate, at which the E-wave and A-wave in the trans-mitral Doppler echocardiography stand adjacent without any overlap. In a conventional group, the dose of ivabradine was adjusted to achieve a heart rate between 50 and 60 beats per minute without using echocardiography. LVEF, left-ventricular ejection fraction. * *p* < 0.05 by Wilcoxon signed-rank test.

Crucially, the analysis unveiled a significant increase in left-ventricular ejection fraction exclusive to the echo-guided group (Figure 8A) [38]. This observation substantiates the substantial benefit of employing echocardiographic guidance in the modulation of heart rate for this specific patient population, although further accumulating studies are warranted to validate the methodology. However, it is acknowledged that the repeated use of echocardiography can pose logistical challenges, primarily due to limitations in clinical resources. As an alternative approach, the formula that the author’s team previously introduced, utilizing the deceleration time to estimate the target heart rate, can be leveraged [11].

An intriguing finding in the echo-guided group was the notable increase in the equivalent dose of carvedilol (Figure 8B) [38]. This phenomenon underscores the hemodynamic stabilization induced by cardiac reverse remodeling, offering an opportune window for the up-titration of neuro-hormonal blockers, including beta-blockers [18]. In this context, ivabradine may exert an auxiliary effect by facilitating the up-titration of neuro-hormonal blockers through its capacity to stabilize hemodynamics. This multifaceted strategy, incorporating echocardiographic guidance and the calculated ideal heart rate, holds promise for optimizing patient care in the realm of heart-failure management. According to overlap therapy, ivabradine therapy may not necessarily be limited to the current guidelines’ indication: those with LVEF ≤ 35% or with resting heart rate ≥ 70 bpm. For example, ivabradine may be indicated even in patients with resting heart rate < 70 bpm if they have obviously overlapped trans-mitral waves. Further studies are warranted to validate these preliminary findings and demonstrate the clinical implication of echo-guided ivabradine therapy in a variety of clinical scenarios.

## 12. Expanding Ivabradine Indications to Other Clinical Scenarios

The sub-analysis of the SHIFT trial has established that heart-rate reduction with ivabradine is both feasible and effective in patients with HFrEF, even across various risk indicators, including those with low systolic blood pressure, low left-ventricular ejection fraction, and high New York Heart Association functional class [39]. This encourages a natural inquiry into the potential clinical implications of ivabradine in settings beyond HFrEF.

One such scenario pertains to cardiac surgery, where the presence of tachycardia has been linked to unfavorable clinical outcomes. A recent study conducted a comparative analysis between two groups: one undergoing heart-rate modulation with ivabradine and the other employing conventional anti-anginal medications alone, without ivabradine, in the preoperative phase of off-pump coronary-artery bypass surgery [40]. In the ivabradine group, ivabradine could be safely integrated with other anti-anginal agents during the preoperative period. This approach effectively maintained lower heart rates during surgery, consequently diminishing the need for beta-blockers, which can potentially exacerbate hemodynamic instability. However, it is noteworthy that there were no discernible differences between the two groups concerning parameters such as troponin T and plasma B-type natriuretic peptide levels at 24 h post-surgery, time to extubation, inotropic support requirements, the incidence of arrhythmias, in-hospital mortality, and 30-day mortality.

These findings suggest that the introduction of ivabradine into the preoperative regimen for cardiac surgery patients could be a valuable strategy for achieving optimal heart rates without compromising post-surgical outcomes. This underscores the potential of ivabradine’s utility in diverse clinical scenarios beyond HFrEF.

## 13. Controversial Aspects of Ivabradine Indication

A nuanced analysis of ivabradine’s potential indications reveals certain areas of controversy. In a meta-analysis encompassing patients with HFpEF [41], ivabradine succeeded in significantly reducing heart rate in this patient cohort. However, this reduction did not yield significant clinical benefits. As discussed above, aggressive heart-rate reduction may inadvertently lead to a decrease in cardiac output in this population. Furthermore, the high prevalence of non-cardiac comorbidities within the HFpEF population might dilute the discernible clinical benefit of heart-rate modulation by ivabradine. Thus, most patients with HFpEF may not have the indication of ivabradine, unless there is obvious overlap between the E-wave and A-wave due to extreme sinus tachycardia.

The suitability of ivabradine’s indication in patients with acute heart failure raises additional concerns. Previous studies have shown that ivabradine can effectively lower heart rate in this context, but it did not yield significant improvements in mid-term mortality and morbidity [42,43]. The optimal heart-rate management strategy during the acute phase remains an unresolved issue. Aggressive heart-rate reduction during this period may indeed reduce cardiac output. A relatively higher heart rate may be required in this clinical scenario to compensate for incremental oxygen demand. Moreover, the acute phase may offer a therapeutic window for up-titrating neuro-hormonal blockers even without ivabradine.

Elevated heart rates have been associated with an increased risk of cardiovascular events in patients with stable ischemic heart disease [44]. Ivabradine has shown the ability to enhance exercise capacity and reduce the frequency and severity of angina symptoms in such cases [45]. Post-hoc analyses of randomized controlled trials even suggested that ivabradine might improve cardiovascular outcomes in patients with stable angina [46]. However, the SYGIFY trial, involving patients with stable angina, failed to corroborate these findings [47]. As of now, the existing evidence does not substantiate ivabradine therapy in patients with coronary-artery disease in the absence of heart failure. Clinical implication of ivabradine in patients with chronic coronary-artery disease and heart failure remains the next concern.

In animal models of sepsis, ivabradine exhibited the capacity to mitigate septic tachycardia, akin to atenolol [48]. Unlike atenolol, ivabradine did not compromise cardiac output or left-ventricular ejection fraction. A randomized controlled trial involving clinical septic shock also reported that ivabradine reduced heart rate, lowered SOFA scores, and increased stroke volume and left-ventricular ejection fraction compared to a placebo [49]. Nevertheless, ivabradine did not exert a significant impact on improving cardiac output or mortality. The present body of evidence does not support ivabradine therapy in patients with sepsis. In this clinical scenario, tachycardia may just be a marker of disease severity and the aggressive approach to this marker may not necessarily improve clinical outcomes. Instead, aggressive intervention to the sepsis itself may be of great importance to improve clinical outcomes.

Several limitations warrant consideration when interpreting these findings. Ivabradine boasts robust evidence in patients with HFrEF [8], yet these studies incorporated patient cohorts with varying left-ventricular ejection fractions. The determination of the optimal heart rate in these diverse clinical scenarios remains an elusive task. The application of the overlap theory may provide a promising avenue to identify ideal heart rates [11]. It is crucial to acknowledge that sinus tachycardia not only represents a therapeutic target but also serves as a marker of underlying severe comorbidities. As such, a comprehensive approach should encompass the identification and management of these comorbidities.

## 14. Future Considerations

Recent investigations have introduced the intriguing possibility of utilizing ivabradine for heart-rate control in patients with atrial fibrillation [50]. While ivabradine primarily blocks the pacemaker current in the sinoatrial node, it has also been suggested that this current is expressed in the atrioventricular node [51]. Consequently, a limited number of studies have explored the use of ivabradine to regulate ventricular rates in patients with atrial fibrillation.

A retrospective study demonstrated an average reduction in ventricular rate from 105 to 89 beats per minute in individuals with permanent atrial fibrillation following ivabradine administration [52]. In another translational clinical trial, ivabradine produced a moderate reduction in heart rate for patients with permanent atrial fibrillation [53]. This heart-rate reduction was primarily attributed to the inhibition of the funny current in the atrioventricular node. In comparison with digoxin, ivabradine proved to be less effective but better tolerated, with a comparable rate of serious adverse events. Ivabradine has no negative impact on hemodynamics and might be clinically useful in patients with supra-ventricular tachyarrhythmias, including atrial fibrillation and any other supra-ventricular tachyarrhythmias, accompanying hemodynamic deterioration. It is important to note that ivabradine therapy for patients with such supra-ventricular tachyarrhythmias is considered off-label, and further translational studies are required to explore its potential indications in this clinical context.

Moreover, ivabradine may offer an avenue for stabilizing hemodynamics and ameliorating hypotension in patients with HFrEF and sinus tachycardia [18]. An increase in blood pressure, although its degree is slight, could provide additional opportunities for the up-titration of anti-heart-failure agents, including beta-blockers. This ivabradine-incorporated therapeutic strategy should be subjected to validation in forthcoming research endeavors, shaping the trajectory of heart-failure management.

## 15. Conclusions

In patients with HFrEF, a higher heart rate has been consistently associated with increased mortality and morbidity. The imperative for up-titrating beta-blockers to their maximum dose is underscored in the management of HFrEF. However, achieving this goal is often challenged by residual congestion, severe cardiac impairment, and hypotension. Enter ivabradine, a recently introduced I_f_ channel blocker renowned for its ability to reduce heart rate without detriment to hemodynamics. The modulation of heart rate with ivabradine not only has the potential to stabilize hemodynamics but also offers a gateway for the further up-titration of anti-heart-failure agents, notably beta-blockers.

It is pivotal to note that the up-titration of ivabradine should be pursued aggressively to ensure a meaningful reduction in heart failure. Paradoxically, excessive heart-rate reduction may inadvertently result in diminished cardiac output and the deterioration of hemodynamics. In this context, the author’s team introduced the concept of the overlap theory. At the optimal heart rate, where cardiac output is maximized, the E-wave and A-wave should ideally stand adjacent without any overlap. This optimal heart rate has demonstrated favorable clinical outcomes across diverse clinical scenarios, including HFpEF. The efficacy of ivabradine therapy appears to be significantly enhanced when trans-mitral Doppler echocardiography is employed as a reference to optimize heart rate.

The subsequent concern revolves around the broader applicability of ivabradine therapy beyond HFrEF. Ivabradine may not exhibit the same level of effectiveness in patients with HFpEF, acute heart failure, sepsis, or stable angina. However, there is a potential avenue for ivabradine in heart-rate modulation for patients with atrial fibrillation, which warrants further exploration. Future studies are necessary to delineate the expanded indications for ivabradine and its role in optimizing patient care.

## Figures and Tables

**Table 1:** Indication of ivabradine for heart-failure patients in each guideline [1,2,3].

	Class
JCS/JHFS guidelines [1]	
Resting heart rate ≥ 75 bpm with sinus rhythm, LVEF ≤ 35%, and refractory to optimal medical therapy	IIa
ESC guidelines [2]	
Resting heart rate ≥ 70 bpm with sinus rhythm, LVEF ≤ 35%, and refractory to optimal medical therapy	IIa
AHA/ACC/HFSA guidelines [3]	
Resting heart rate ≥ 70 bpm with sinus rhythm, LVEF ≤ 35%, and refractory to optimal medical therapy	IIa

JCS, Japanese Circulation Society; JHFS, Japanese Heart Failure Society; ESC, European Society of Cardiology; AHA, American Heart Association; ACC, American College of Cardiology; HFSA, Heart Failure Society of America; LVEF, left-ventricular ejection fraction.
